# Rapid Capture and Analysis of Airborne *Staphylococcus aureus* in the Hospital Using a Microfluidic Chip

**DOI:** 10.3390/mi7090169

**Published:** 2016-09-15

**Authors:** Xiran Jiang, Yingchao Liu, Qi Liu, Wenwen Jing, Kairong Qin, Guodong Sui

**Affiliations:** 1 Department of Biomedical Engineering, Dalian University of Technology, Dalian 116024, China; xrjiang@dlut.edu.cn (X.J.); krqin@dlut.edu.cn (K.Q.); 2 Department of Neurosurgery, Provincial Hospital Affiliated to Shandong University, Jinan 250021, China; bxs103@sdu.edu.cn; 3 Department of Environmental Science & Engineering, Fudan University, Shanghai 200433, China; 13110740002@fudan.edu.cn (Q.L.); jingwenwen1983@gmail.com (W.J.)

**Keywords:** microfluidics, airborne bacteria, bioaerosols

## Abstract

In this study we developed a microfluidic chip for the rapid capture, enrichment and detection of airborne *Staphylococcus* (*S.*) *aureus*. The whole analysis took about 4 h and 40 min from airborne sample collection to loop-mediated isothermal amplification (LAMP), with a detection limit down to about 27 cells. The process did not require DNA purification. The chip was validated using standard bacteria bioaerosol and was directly used for clinical airborne pathogen sampling in hospital settings. This is the first report on the capture and analysis of airborne *S. aureus* using a novel microfluidic technique, a process that could have a very promising platform for hospital airborne infection prevention (HAIP).

## 1. Introduction

*Staphylococcus* (*S.*) *aureus*, which is one of the major community-acquired pathogens, has been reported to be responsible for various human diseases [1]. Further, *S. aureus* has the ability to colonize the human nose and wound area on the skin, causing hospital airborne infections [2]. Due to the lack of a technique for rapidly detecting airborne *S. aureus*, bacterial transfer by air is hard to prevent and has become a serious threat to public safety and patients in hospital wards. At present, few techniques are capable of rapid detection of airborne *S. aureus*.

In the field of airborne pathogen detection, several traditional techniques have been proposed, such as the Anderson sampler and all-glass impinger (AGI) sampler [3]. However, the pathogen concentrations in samples collected and recovered by these techniques are too low for direct bioanalysis [4,5]. Furthermore, these techniques all require an obligatory culturing step, which is the most time-consuming stage of the analysis, as this usually takes days to complete [6]. Moreover, most of the known pathogens that exist in the natural environment are in a viable but non-culturable (VBNC) state [7], thus they cannot be detected using a culture method. So rapid bioanalysis of airborne pathogen is very hard to perform, and this is the biggest bottleneck for current sampling techniques. As a result, the limited techniques have made it difficult to issue a pre-warning of airborne *S. aureus*–related diseases.

The efficient transfer of pathogens from air to favorable media is the most essential step [7] for a rapid analysis technique. Another vital factor is that the result of the analysis is easily visualized, without the need for extensive or elaborate processing such as conventional electrophoresis or hybridization. Loop-mediated isothermal amplification (LAMP) is an attractive technique with high sensitivity and selectivity, as well as less processing time [8] compared with traditional molecular analysis methods such as polymerase chain reaction (PCR). More importantly, the LAMP reaction can be performed under isothermal conditions and the result is visible to the naked eye under a 365 nm ultraviolet (UV) lamp, without the need for electrophoresis, which makes it a very convenient step for a downstream bioanalytical technique [9]. Although bioanalysis integrated with LAMP has been used for the detection of microorganisms, LAMP analysis of airborne samples has rarely been referred to.

Microfluidic-based techniques have recently drawn lots of attention because of their low reagent consumption, short analysis time and environmentally friendly process which can be used for developing portable biosensors [6]. Pathogen analysis using microfluidic chips has been frequently reported, but few studies have involved the airborne analysis of *S. aureus*. A microfluidic system with a staggered herringbone mixer (SHM) structure that is capable of rapid and efficient capture and enrichment of airborne bacteria was established previously in our lab [10]. Based on a similar technique, we report in this study the rapid capture and enrichment of airborne *S. aureus*, followed by direct LAMP analysis. Compared to the conventional Anderson sampler that has a collection and analysis time of several hours and days, respectively, the chip described in this study significantly decreased the collection (3 h and 30 min) and analysis time (40 min), and the device was much simpler and more portable. Further, for hospital airborne infection prevention (HAIP) purposes, we were the first to evaluate an airborne pathogen in a hospital environment using the microfluidic technique.

## 2. Materials and Methods

### 2.1. Bacteria and Reagents

*Staphylococcus aureus* was isolated from clinical samples taken from Neweast Hospital (Shanghai, China) and cultured in Luria-Bertani (LB) medium at 37 °C.

### 2.2. Chip Fabrication

The double-layer microfluidic chip was fabricated following the standard soft lithography described previously [11,12]. Two pieces of the silicon molds, including the upper fluidic layer and the bottom staggered herringbone layer, were constructed from 20 μm high SU-8 2025 photoresist (Microchem, Westborough, MA, USA). The staggered herringbone mixer (SHM) structure was designed based on previous work [10]. Two polydimethylsiloxane (PDMS) layers were bonded through baking at 80 °C for 12 h to give a radial chip with 18 s-shaped airborne bacteria capture channels. Access holes of 1.5 mm diameter were drilled along the edge of the round chip of each channel to be used as inlet. Meanwhile, a 3.5 mm diameter hole was drilled in the center of the chip for air flow and bacteria-capture outlet, connecting the 18 airborne bacteria-capture units.

### 2.3. Airborne Staphylococcus aureus Capture and Enrichment

An overnight culture of *S. aureus* suspension was diluted to different concentrations to generate a bioaerosol using an aerosol generator in a 125 L cube tank referred to in our previous studies [10,13]. The chip was placed in the tank and connected to a pump to facilitate the airborne bacteria capture and enrichment.

For the limit of detection (LOD) evaluation, two chips were used in a parallel manner in the experiment. One of the chips was for collecting and counting the captured airborne bacteria, whereas the other one was used for bacterial capture and analysis. An LB culture dish was placed on the bottom of the tank as a parallel control. The pump process has been referred to in our previous work [10,13], but the chip vacuum time being extended to 3 h and 30 min. Following the process of enrichment, the counting chip was washed with ddH_2_O to flush the SHM channels. The washed bacterial cells were then collected with a pipette and transferred to a 1.5 mL tube for counting using the dilution-plate counting method [10]. As for the capture chip, it was washed with 0.5 μL lysis buffer (DEAOU Biotechnology, Dalian, China) per channel in the same manner as for the counting chip, with the difference being that the collected suspension was maintained at room temperature for 30 min to allow lysis of bacterial cells to occur. The cell lysate was then used for direct LAMP analysis without any purification process.

### 2.4. LAMP Reaction System for Nuc Gene Detection

The lysed bacterial suspension was mixed with LAMP reagent (DEAOU Co., Dalian, China) consisting of 0.8 μM each of the inner primer (FIP and BIP), 0.4 μM each of the loop primer (LF and BF), 0.2 μM each of outer primer (F3 and B3), 8U *Bst* DNA Polymerase and 12.5 μL Reaction Mix provided in the kit. The species-specific primer sequences (a total of four primers) of the *nuc* gene (Gene Accession no. V01281) were as described in our previous work [14], and synthesized by Invitrogen (Shanghai, China). LAMP amplification was performed for 40 min in a 65 °C water bath, followed by fluorescence detection under UV excitation at 365 nm.

### 2.5. Clinical Airborne Staphylococcus aureus Analysis

Clinical airborne samples were obtained from six different settings in Shandong Hospital, including the intensive care unit (ICU), surgery room, emergency room, surgical ward, outpatient service hall and doctor’s office. The radial chip was placed on a well-ventilated site for airborne sample capture. The vacuum time was set as 3 h and 30 min, followed by washing of the channels and LAMP analysis.

## 3. Results and Discussion

### 3.1. Chip Design

**Figure 1:**
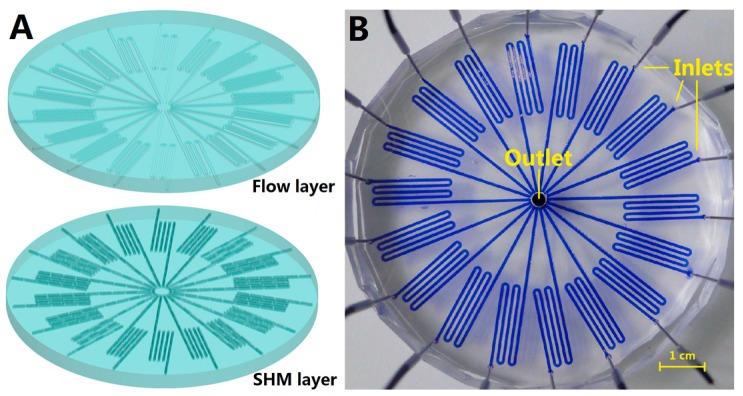
Schematic illustration of the radial airborne bacteria capture chip. (**A**) Schematic illustration of the assembly of the microfluidic chip; (**B**) Top view of the radial chip, showing 18 inlets along the outer edge of each channel for air inlet and a 3.5 mm diameter hole in the center for air and a washed solution outlet. The channels were loaded with blue dye to show the clear structure.

The microfluidic chip with 18 SHM channels is shown in Figure 1. The chip had a diameter of about 7 cm, and each of the channels had a height of 40 μm and a width of 600 μm. To facilitate the capture and enrichment of airborne bacteria, the 18 channels within the chip in the bottom layer contained the SHM structure for capturing the airborne bacteria, while the upper layer contained 18 flow channels at the corresponding position. The chip was assembled from the SHM layer and flow layer, both fabricated from polydimethylsiloxane (PDMS). Eighteen access holes along the edge of the round chip were used as airflow inlets. The central hole was used for air flow and bacteria-capture outlet.

To facilitate the capture of airborne bacteria, a vacuum was connected to the center of the radial chip via a 3.5-mm-diameter stainless steel tube, drawing the bacterial aerosol into the channels from the holes along the outer edge of the chip under the negative force created by the vacuum. By breaking the laminar flow to twisted air flow inside the channel, the contact opportunity between the channel wall and the bacteria in the airflow is increased, so the chip can collect airborne bacteria with very high efficiency [7,10]. The number of SHM channels within the chip was increased to 18 so as to increase the total amount of air that would pass through the channels.

### 3.2. Detection Limit of Staphylococcus aureus in the Microfluidic Chip

We evaluated the utility of the microfluidic method using standard bacterial bioaerosols in a 125 L cube tank. *S. aureus* was chosen because it is frequently associated with hospital airborne infectious diseases. The sensitivity of the microfluidic chip was validated by *S. aureus* bioaerosols using overnight cultured bacteria ranging from 1.92 × 10^2^ to 1.92 × 10^−1^ CFU/mL. The plate sedimentation method was also performed in parallel for comparison with the chip method.

**Figure 2:**
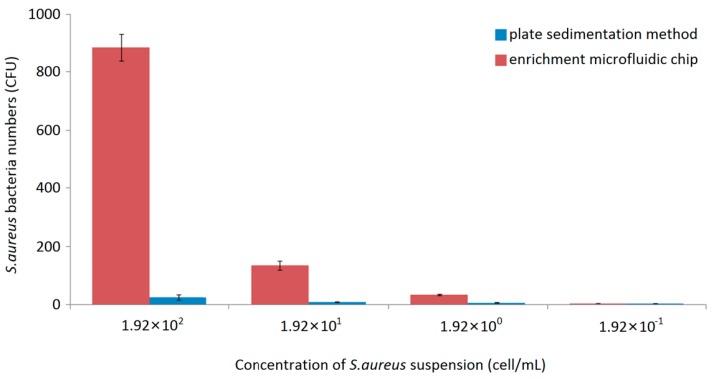
*Staphylococcus* (*S.*) *aureus* cells collected by the plate sedimentation method or captured by the microfluidic chip.

The airborne *S. aureus* in the bioaerosol was successfully captured by using the microfluidic chips containing the SHM channels. Figure 2 compares the number of *S. aureus* that was collected by the radial chip and the plate sedimentation method. Each experiment was performed three times and the average number was used.

Compared to our previous data [5], the extent of the vacuum time significantly increased the ratio of the number of chip-captured bacteria to the number of plate-collected bacteria. The reason might be the increased number of the capture channels, which greatly increased the air flow passing through the SHM structures, thus capturing more bacteria within the chip.

When the concentration of the *S. aureus* suspension was 10^2^ cell/mL, approximately 885 cells (average number) were captured by the chip, which was about 42 times higher than that collected by the plate sedimentation method (21 cells). When the bacterial concentration was decreased to 10 cell/mL, 142 cells were captured by the chip compared to about five cells collected by the plate sedimentation method. Further reduction of the *S. aureus* concentration to 1 cell/mL, although a very low cell concentration, still resulted in 27 cells being captured by the chip, compared to one cell collected by the plate sedimentation method. When the bacterial suspension was used at a concentration of 10^−1^ cell/mL, only about one cell was captured both by the chip method and the plate sedimentation method.

**Figure 3:**
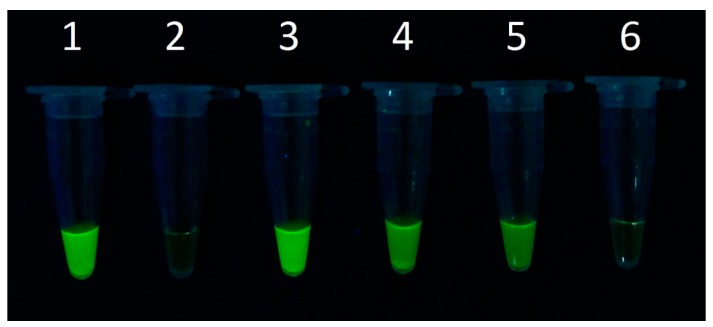
Loop-mediated isothermal amplification (LAMP) analysis of the system sensitivity using diluted *S. aureus*. (**1**) *S. aureus* DNA; (**2**) ddH_2_O; (**3**) LAMP product of about 885 cells collected by the chip; (**4**) LAMP product of about 142 cells; (**5**) LAMP product of about 27 cells; (**6**) LAMP product of about one cell.

The chip-captured bacteria were washed and used as samples for direct LAMP analysis. The *nuc* gene was chosen for the LAMP analysis, because the gene encodes a thermostable nuclease that is found only in *S. aureus*, and thus can be used as a specific target for the detection of *S. aureus* [5,15]. Positive LAMP results were obtained when the numbers of chip-captured bacteria were equivalent to approximately 885, 142 and 27 cells (Figure 3). Further reduction of the collected cells to about one cell did not yield any detectable signal. Therefore, the detection limit for the system was about 27 CFU for *S. aureus*.

### 3.3. Clinical Airborne Staphylococcus aureus Analysis

**Figure 4:**
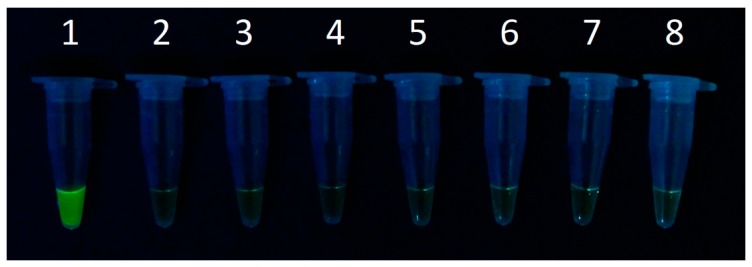
LAMP analysis of airborne bacteria collected in Shandong Hospital. (**1**) *S. aureus* DNA; (**2**) ddH_2_O; (**3**) Airborne bacteria samples from intensive care unit (ICU); (**4**) Surgery room; (**5**) Emergency room; (**6**) Surgical ward; (**7**) Outpatient service hall; (**8**) Doctor’s office.

Six different wards in Shandong Hospital were chosen for airborne sample collections. The collected samples were directly subjected into LAMP assay without a conventional DNA extraction step. As shown in Figure 4, the LAMP results for the samples collected from the hospital were negative, indicating that the number of bacteria collected was lower than our LOD (about 27 cells).

This is the first report to describe the utilization of a microfluidic chip for airborne bacteria analysis in the hospital. Although traditional techniques for the detection of airborne bacteria have been used since 1881 [7], there is still a gap between collecting the bacterial sample and direct diagnosis, and the reason is that the bacterial concentration of the collected and recovered samples is too low for direct bioanalysis, such as PCR or immunoanalysis. Current sampling techniques could not solve the problem of bacterial concentration, while the culturing step needed for the identification of the microorganisms usually takes days to complete, which is not ideal for the early diagnosis of diseases.

Furthermore, there is still no technique capable of accurately evaluating the distribution of pathogens in the air, which is a critical piece of data for addressing the concern of hospital infection as it enables the indoor air quality in a hospital to be assessed. Based on the published data using an Anderson sampler, the airborne bacterial concentration is approximately 1 × 10^3^ to 6 × 10^3^ CFU/m^3^ in an ordinary hospital [16]. The total number of viable cells was greatly underestimated, considering that some of the bacteria might have been killed by the strong impact at which the cells strike the culture plate during the Anderson sampling process. The collected dead bacteria that were distributed in the air would not grow on a culture plate. Besides, many pathogen species are in a viable but non-culturable state (VBNC) in the natural environment [4]. Thus, the number of bacteria that would finally grow on the culture plate could not really account for the actual number of bacteria originally present in the air. Although the Anderson sampling method is limited as discussed above, there has been no other technique that can be used in parallel to verify the amount of bacteria in the air.

The microfluidic chip that we presented in this paper could capture both living and dead bacteria, as well as DNA fragments suspended in the air. During the wash step, the bacteria along with the DNA fragments were all mixed in the solution. Thus, the microfluidic chip can collect a huge number of bacteria within a couple of microliters of aqueous medium, so that the bacterial concentration would be high enough for direct bioanalysis. By obtaining samples of airborne bacteria with a high concentration, molecular bioanalysis can be carried out as soon as the sampling process is completed, and this was considered as a great progress in the field of the rapid analysis of airborne pathogens in the hospital.

However, having an effective amount of air to pass through the channels is still the bottleneck of the microfluidic chip technique. In our study, to increase the amount of air passing through the chip channels, the air flow was set at 79.2 mL/min, and this was almost the highest air flow (mL/min) that could be obtained for a normal-sized microfluidic chip, given the necessary channel length should be longer than 17.4 cm to ensure no airborne cells would leak from the chip [10]. After 3 h and 30 min of the vacuum process, the amount of air flow that passed through the chip channels was estimated to be about 16.63 L, a very large amount of air for a normal-sized microfluidic chip. In order to further increase the air flow, a longer vacuum time and more pumps are needed.

The negative result obtained for the hospital airborne sample analysis showed that the number of captured *S. aureus* was lower than the LOD of the microfluidic chip (27 cells). Nevertheless, the microfluidic chip showed that the concentration of *S. aureus* in the hospital air was lower than 1.6 cells per liter of air (27 cells divided by 16.63 L), a result that was not possible to obtain with any existing traditional techniques. On the basis of this, our work may provide a potential platform for airborne pathogen sampling and bioanalysis, especially in the prevention of hospital airborne pathogen infections.

## 4. Conclusions

Few studies have been conducted to evaluate airborne *S. aureus*. We have successfully demonstrated a radial airborne bacteria capture and enrichment chip for fast assessment of airborne *S. aureus*. The system could perform airborne *S. aureus* capture, enrichment, and rapid LAMP analysis. The bacteria were collected by the radial chip with an SHM structure. They were washed and then directly subjected to LAMP analysis without the need for DNA purification. Standard *S. aureus* bioaerosol was used to validate the system. A detection limit down to approximately 27 cells was achieved for *S. aureus*. The presented microfluidic technique for rapid capture and analysis of airborne *S. aureus* could have a huge potential in disease control and clinical applications, making it a promising for future point-of-care tests in the field, especially given its unique properties compared to traditional techniques.
